# Differential analysis of microRNAs in plasma exosomes in patients with cerebral ischemic stroke

**DOI:** 10.1097/MD.0000000000040677

**Published:** 2024-11-29

**Authors:** Haifang Lan, Shengshan Yuan, Yanlun Song, Tingjun Liu, Gautam Agarwal, Xuebin Li, Lina Liang, Guijiang Wei

**Affiliations:** a Center for Medical Laboratory Science, Affiliated Hospital of Youjiang Medical University for Nationalities, Guangxi, China; b Baise Key Laboratory for Research and Development on Clinical Molecular Diagnosis for High-Incidence Diseases, Guangxi, China; c Key Laboratory of Research on Clinical Molecular Diagnosis for High Incidence Diseases in Western Guangxi, Guangxi, China; d Department of Neurology, Affiliated Hospital of Youjiang Medical University for Nationalities, Guangxi, China; e Modern Industrial College of Biomedicine and Great Health, Youjiang Medical University for Nationalities, Guangxi, China.

**Keywords:** cerebral ischemic stroke, exosomes, gene regulatory networks, high-throughput nucleotide sequencing, microRNA

## Abstract

The high incidence, disability, mortality, and recurrence rates of cerebral infarction impose a heavy burden on both the Chinese and global populations. It is essential for the early diagnosis, prevention, and protection against brain cell injury. To identify differentially expressed microRNAs (miRNAs) in plasma exosomes of patients with cerebral ischemic stroke, determine relevant biomarkers, and explore their potential signaling pathways. High-throughput sequencing was used to detect the expression of plasma exosomal miRNAs in patients with cerebral ischemic stroke and in a control group. Gene Ontology, Kyoto Encyclopedia of Genes and Genomes enrichment, and target gene network analyses were performed to investigate the target genes and signaling pathways of the differentially expressed miRNAs. The sequencing results identified 95 differentially expressed miRNAs, with 40 upregulated and 55 downregulated miRNAs. Among these, hsa-miR-1303, hsa-miR-125b-1-3p, and hsa-miR-548ab were significantly upregulated in the stroke group and downregulated in the normal control group, whereas hsa-miR-1289 was downregulated in the stroke group and upregulated in the normal group. Gene Ontology, Kyoto Encyclopedia of Genes, and genomes enrichment analyses indicated that the differentially expressed miRNAs and their target genes were mainly concentrated in the PI3K-AKt, mitogen-activated protein kinase, calcium, Ras, Rap1, and cAMP signaling pathways. The expression of plasma exosomal hsa-miR-1303, hsa-miR-125b-1-3p, and hsa-miR-1289 was significantly different in stroke patients than in the control group. These miRNAs may be involved in various signaling pathways related to cerebral infarction, providing a reference for further experimental research.

## 1. Introduction

Cerebral ischemic stroke (CIS) is a major public health concern worldwide, being the second leading cause of death and third leading cause of disability. This imposes a significant burden on healthcare systems, especially in China, where the prevalence of stroke risk factors such as hypertension, diabetes, and an aging population is increasing.^[1,2]^ CIS occurs when the blood flow to a part of the brain is obstructed, leading to tissue damage and loss of neurological function. The prognosis of CIS patients depends on the initial severity of the neurological deficit, age, and comorbidities such as hypertension or diabetes mellitus.^[2]^ Prompt diagnosis and intervention are essential to improve outcomes and reduce the long-term disabilities associated with CIS.

Recent advancements in the study of exosomes have opened new avenues for the diagnosis and treatment of various diseases including CIS. Exosomes are nano-sized extracellular vesicles secreted by a variety of cells, including those in the central nervous system. They play a significant role in cell communication by transporting complex biomolecules, such as proteins, lipids, and nucleic acids, which can influence the behavior of recipient cells.^[3,4]^ In view of the characteristics of exosomes, this study investigated changes in the peripheral blood of patients after acute cerebral infarction to understand the biomolecular changes in the central nervous system.

MicroRNAs (miRNAs) are a class of small noncoding RNAs that regulate gene expression at the posttranscriptional level. They are involved in numerous biological processes (BP) including cell proliferation, differentiation, and apoptosis.^[5]^ miRNAs carried by exosomes are protected from degradation in bodily fluids, allowing them to serve as stable biomarkers for disease diagnosis and prognosis.^[6–8]^ In CIS, miRNAs in plasma exosomes have demonstrated potential as noninvasive biomarkers for early detection and monitoring of the disease.

Despite the growing body of research on exosomal miRNAs in CIS, most studies have focused on animal models, with limited data available from human subjects. This study aimed to address this gap by analyzing the expression profiles of miRNAs in plasma exosomes from patients with CIS in Guangxi, China. Using high-throughput sequencing, we identified differentially expressed miRNAs and conducted bioinformatic analyses to explore their potential roles in CIS pathogenesis. Our findings provide a foundation for further research on exosomal miRNAs as biomarkers for CIS and may contribute to the development of new diagnostic and therapeutic strategies.

## 2. Materials and methods

### 2.1. Patient recruitment

Patients who met the diagnostic criteria for CIS^[9]^ within 48 hours of onset and had no other serious diseases were included in the case group. Recruit hospitalized non-CIS patients of similar age, sex, and underlying diseases to the control group. All the recruited patients voluntarily signed an informed consent form and were recruited from Guangxi, China. Clinical data comparing hypertension, diabetes, homocysteine, fasting blood glucose, total cholesterol, triglycerides, high-density lipoprotein cholesterol, low-density lipoprotein cholesterol, and coagulation function, between the 2 groups of patients are detailed in Table 1.

**Table 1:** Basic clinical information between case group and control group.

Number	Gender	Age	Hypertension	Diabetes	HCY (µmol/L)	GLU (mmol/L)	TC (mmol/L)	TG (mmol/L)	HDL-C (mmol/L)	LDL-C (mmol/L)	Blood clotting functions
100905	F	61	Y	N	9.5	7.24↑	3.34	1.10	0.92	2.39	Normal
100906	F	68	Y	N	11.6	10.17↑	5.61	1.85↑	1.08	3.74↑	APTT-T↓
100801	M	55	Y	N	15.00	5.21	4.20	2.01↑	1.08	2.76	APTT-T↓
100904	M	57	Y	N	11.40	4.94	4.22	1.07	1.02	2.54	Normal
101101	F	58	Y	N	8.4	4.27	6.29↑	1.37	1.63	3.96↑	PT-T↓
101301	M	69	Y	N	9.50	5.14	4.49	1.612	0.66↓	3.12	Normal

*Note:* 100905, 100906, and 100801 are the case group and 100904, 101101, and 101301 are the control group. F = female, GLU = glucose, HCY = homocysteine, HDL-C = high-density lipoprotein cholesterol, LDL-C = low density lipoprotein cholesterol, M = male, N = no, Y = yes.

### 2.2. Plasma specimen collection and extraction of plasma exosomes

On the morning of the second day after admission, 10 mL of fasting venous blood was collected from each of the 3 patients in the case group and 3 patients in the control group, and placed in EDTA tubes. The blood was then centrifuged at 3000 × g for 30 minutes at 4 °C, and 5 mL of the upper plasma was collected and frozen at −80 °C for future use. When extracting exosomes, the plasma samples were rapidly thawed in a water bath at 37 °C and transferred to a new centrifuge tube. The samples were then centrifuged at 2000 × g for 30 minutes at 4 °C. The upper clear solution was transferred to a new centrifuge tube and centrifuged at 10,000 × g for 45 minutes at 4 °C to isolate medium or small vesicles. The upper clear liquid was removed, filtered using a 0.45 μm filter membrane, and the filtrate was collected. Subsequently, the filtrate was transferred to a new centrifuge tube and centrifuged at 10,000 × g for 70 minutes at 4 °C. Finally, the resulting filtrate was transferred to another centrifuge tube and centrifuged at 10,000 × g for 70 minutes at 4 °C to obtain the plasma exosomes.

### 2.3. Identification of exosomes

Plasma exosomes were rapidly thawed at 37 °C. Next, 10 μL of exosomes were deposited onto copper grids for 1 minute, followed by absorption of excess liquid with filter paper. Then, 10 μL of uranium acetate was added to the copper grids for 1 minute, and the excess solution was absorbed with filter paper before air-drying at room temperature for several minutes. Transmission electron microscopy (Hitachi, HT-7700, Japan) was performed at 100 kV. The diameter distribution of exosomes was assessed via nanoparticle tracking analyzer (Particle Metrix, PMX120, Germany).^[3]^

### 2.4. Extraction of total RNA from plasma exosomes

After the sample was retrieved from the -80 °C refrigerator, 250 μL to 1 mL samples were used for extraction, with only 250 μL plasma samples extracted per tube. The number of separate tubes was determined on the basis of the extraction volume. For example, using a 250 μL sample, 750 μL of TRIzol LS Reagent was added to 250 μL of the sample, mixed well, and incubated for 5 minutes at room temperature. Next, 250 μL of chloroform was added, fully mixed for 15 seconds, and incubated for 2 to 3 minutes at room temperature. The mixture was centrifuged at 12,000 rpm at 4 °C for 15 minutes. The supernatant was carefully transferred to a new 1.5 mL centrifuge tube. An equal volume of isopropanol and 1 μL of glycogen were added, mixed well by inversion, and incubated at −80 °C for 1 hour. Then, the mixture was centrifuged at 12,000 rpm and 4 °C for 30 minutes. The supernatant was discarded and washed with 1 mL of precooled 75% ethanol by inverting several times to suspend the precipitate in the liquid. The mixture was centrifuged at 12,000 rpm at 4 °C for 10 minutes. Repeat the washing step; after the final wash, discard the supernatant, and after a short centrifugation, remove any residual ethanol at the bottom with a pipette gun. The tubes were placed in a fume hood for 10 to 20 minutes to air-dry. Ten microliters of RNase-free water were added, mixed well, and dissolved for 1 to 2 minutes. Appropriate samples were collected for quality inspection, and the remaining samples were stored at −80 °C.

Total RNA quality detection: high-quality RNA is essential for library construction. In this study, the RNA quality was assessed using the following method:

(a) Concentration and total RNA amount: quantified precisely using Qubit;
(b) RNA purity: measured by the OD260/280 and OD260/230 ratios using Nanodrop;
(c) RNA integrity: evaluated by agarose gel electrophoresis and Agilent 2100 Bioanalyzer to determine the extent of RNA degradation.

RNA samples with concentrations ≥ 100 ng/μL, total amounts ≥ 2 μg, OD260/280 values between 1.8 and 2.2, OD260/230 values ≥ 2.0, and RIN ≥ 7 (as detected by Agilent 2100 Bioanalyzer) were required to ensure the construction of high-quality downstream small RNA-seq libraries.

### 2.5. Sequencing of microRNAs in plasma exosomes

Millions of miRNA sequences were simultaneously obtained using high-throughput sequencing methods based on the Illumina HiSeq miRNA-seq. This enables the rapid identification of known and unknown miRNAs along with their expression differences in disease states. The specific method flow is as follows:

(a) Ligate 3’ Adapters: the RNA 3’ Adapter was mixed with total RNA and denatured at 70 °C to unfold the secondary structure of RNA. Next, using T4 RNA Ligase 2 (truncated T4Rnl2), the 5’ terminal preadenosine was specifically modified and linked to the 3’ terminal of the target RNA at 25 °C for 1 hour.
(b) Primer hybridization: reverse transcription primers were added and annealed with the 3’ connector to reduce the number of free 3’ connectors directly connected to the 5’ connector in a subsequent step.
(c) Ligate 5’ Adapters: after denaturing the RNA 5’ Adapter at 70 °C, it was added to the previous reaction system and subjected to T4 RNA Ligase action at 25 °C for 1 hour.
(d) Reverse transcription for 1st strand cDNA: the first cDNA strand was synthesized using reverse transcriptase primers complementary to the sequences at the 5’ and 3’ junctions under the action of reverse transcriptase.
(e) PCR amplification and index: the product from the previous step served as a template for synthesizing and amplifying the double-stranded library of miRNAs through PCR. This process specifically enriched miRNAs with splices at both ends. Additionally, 1 side of the PCR primers contained 6-base special tags (Indexes) that were used to distinguish between different samples, thus facilitating the mixing of various libraries. The number of amplification reaction cycles was strictly controlled to minimize the bias introduced by PCR.
(f) PAGE and purification of microRNA library: high-resolution polyacrylamide gel electrophoresis (PAGE) was employed to isolate microRNA libraries with inserts sized between 22 and 24 nt. A 6% PAGE gel was prepared and a Custom Ladder or High-Resolution Ladder was used as the standard. RNAs of different fragment sizes were separated by electrophoresis. Diffuse products corresponding to 140 to 160 bp (with the main miRNA libraries concentrated at approximately 147 bp) were excised under ultraviolet light. The PAGE blocks were then broken by centrifugation and the library products were recovered by soaking.
(g) Library quality check: the constructed sequencing library was checked and quantified. Qubit was used to accurately quantify the library concentration, whereas the Agilent 2100 Bioanalyzer was used to determine the library fragment size distribution and assess its suitability for computer applications.
(h) Sequencing: qualified samples after dilution according to the sample sequencing flux requirements were loaded onto the sequencing machine in accordance with the corresponding molar ratio for multiple samples. The library was sequenced using the Illumina Novaseq high-throughput sequencing platform employing the Qualcomm sequencing strategy.

## 3. Results

### 3.1. Confirmation of exosomes

**Figure 1.**
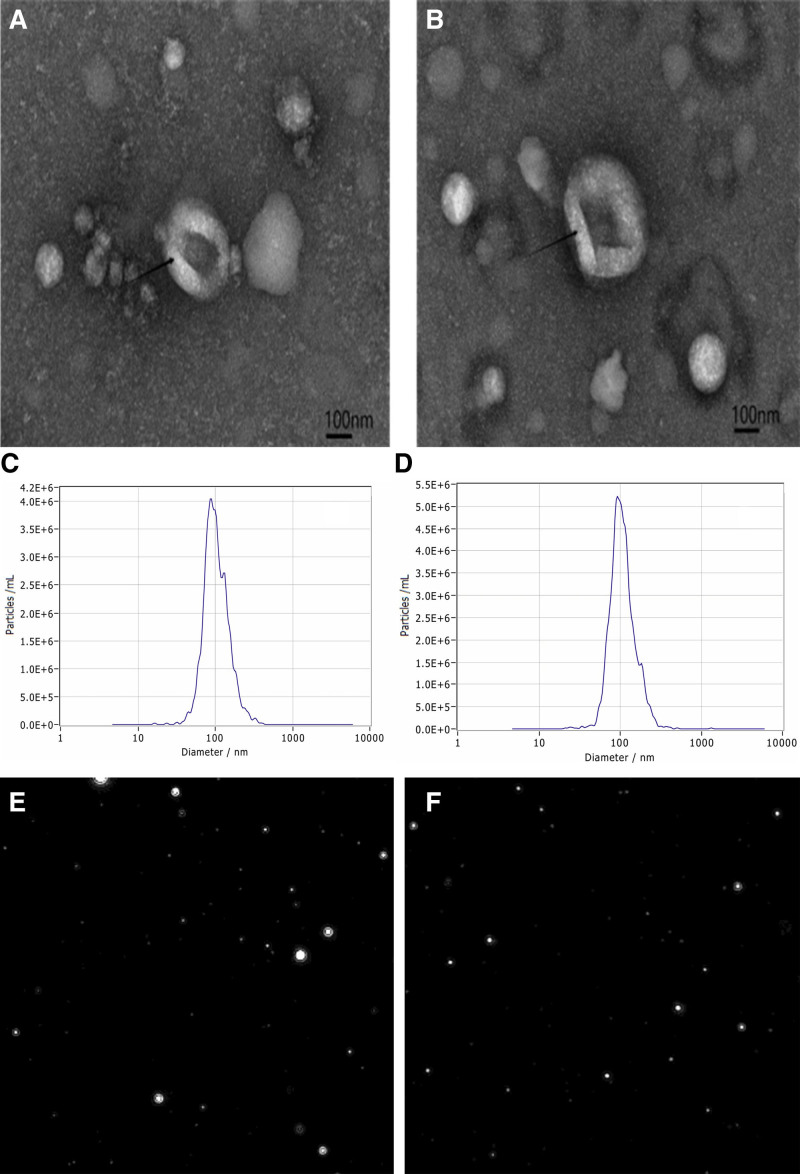
Exosome electron micrograph and nanoparticle tracking analyses. (A, C, and E) Exosome vesicles isolated from the control group. (B, D, and F) Exosome vesicles isolated from the case group.

Multiple circular vesicles with lipid bilayers ranging from approximately 50 to 150 nm in diameter were observed under an electron microscope, all of which exhibited the typical biological characteristics of exosomes (Fig. 1A and B).^[3]^ The size and concentration of exosomes were characterized using nanosight particle tracking analysis, more than 80% of which were between the peaks of 60 and 150 nm in diameter, indicating that the purity and concentration of exosomes met the experimental requirements (Fig. 1C–F).^[10]^

### 3.2. Expression of microRNAs in exosomes

**Figure 2.**
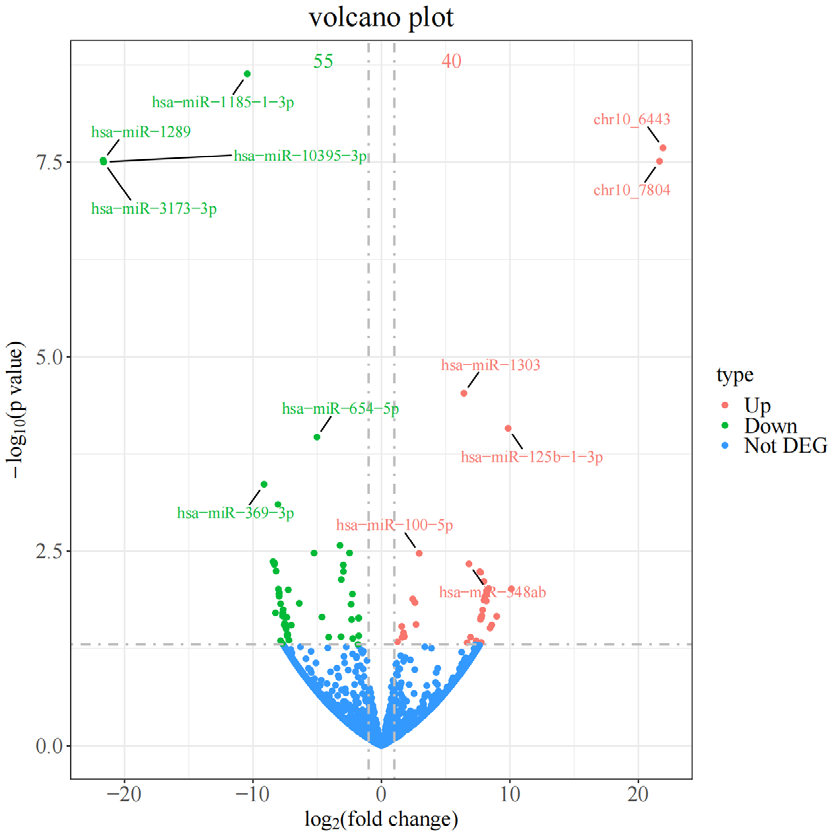
MicroRNAs volcano plot of exosome differences in CIS patients relative to the controls. Red dots indicate genes that are upregulated in the case sample relative to the control sample, green dots indicate genes that are downregulated, and blue dots indicate genes that do not differ significantly.

By comparing the miRNA expression profiles of the CIS and control groups, 95 differentially expressed miRNAs were identified using the DESeq2 software, meeting the criteria of |log2(Fold Change)| > 1 and *P* < .05. Of these, 40 were upregulated and 55 were downregulated. The miRNA volcano plot of exosome differences in patients with CIS relative to the controls is shown in Figure 2. The volcano plot showed that chr10-6443, chr10-7804, hsa-miR-1303, hsa-miR-125b-1-3p, hsa-miR-100-5p, and hsa-miR-548ab were significantly upregulated, whereas hsa-miR-1289, hsa-miR-1185-1-3p, hsa-miR-10395-3p, hsa-miR-3173-3p, hsa-miR-654-5p, and hsa-miR-369-3p were significantly downregulated.

### 3.3. Functional clustering analysis of the screened microRNA differential genes in exosomes

**Figure 3.**
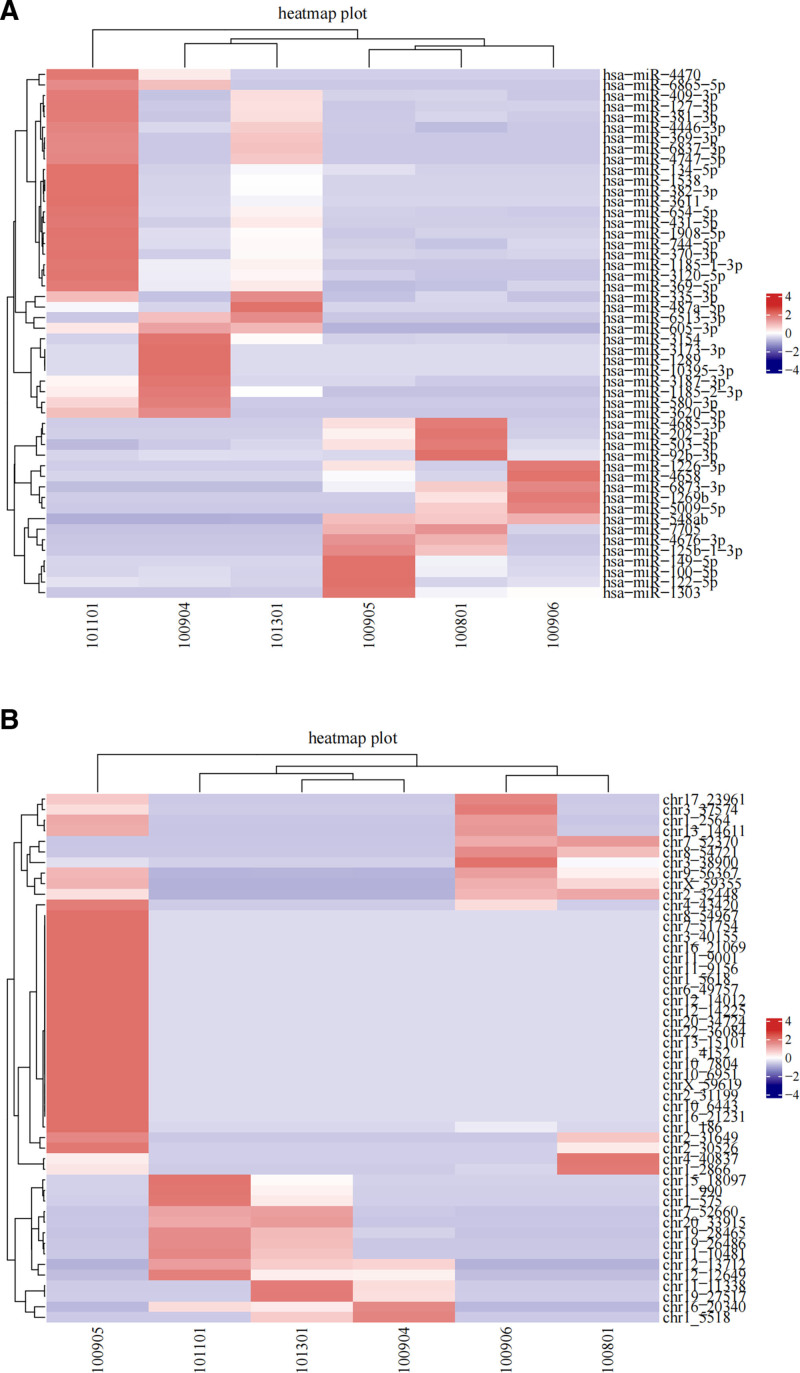
Cluster analysis of named and unnamed microRNAs differentially expressed in exosomes. (A) Named microRNAs; (B) unnamed microRNAs. Each column represents a different sample (100905, 100906, 100801 are the case group, and 100904, 101101, 101301 are the control group). Lines represent different genes, and the corresponding color represents the level of expression of the gene in the sample. Red indicates that the gene is highly expressed in the sample, and green indicates that the gene has low expression in the sample.

Functional clustering analysis of the differentially expressed miRNAs in exosomes was performed to analyze the biological functions of the same or similar miRNAs (Fig. 3). As shown in panel A of Figure 3, hsa-miR-548ab was highly expressed in the CIS group but was low in the control group, whereas hsa-miR-6873-3p was highly expressed in the case group and was significantly lower in the control group. Additionally, hsa-miR-605-3p was highly expressed to varying degrees in the control group and was significantly lower in the case group. As shown in panel B, chr9-56367, chrX-59355, and chr2-32448 were highly expressed to varying degrees in the case group and were significantly lower in the control group, whereas chr12-13712 and chr16-20340 were highly expressed to varying degrees in the control group and were significantly lower in the case group. Furthermore, chr12-12649 was found to be weakly expressed in the case group.

### 3.4. Differential miRNAs target genes prediction

**Figure 4.**
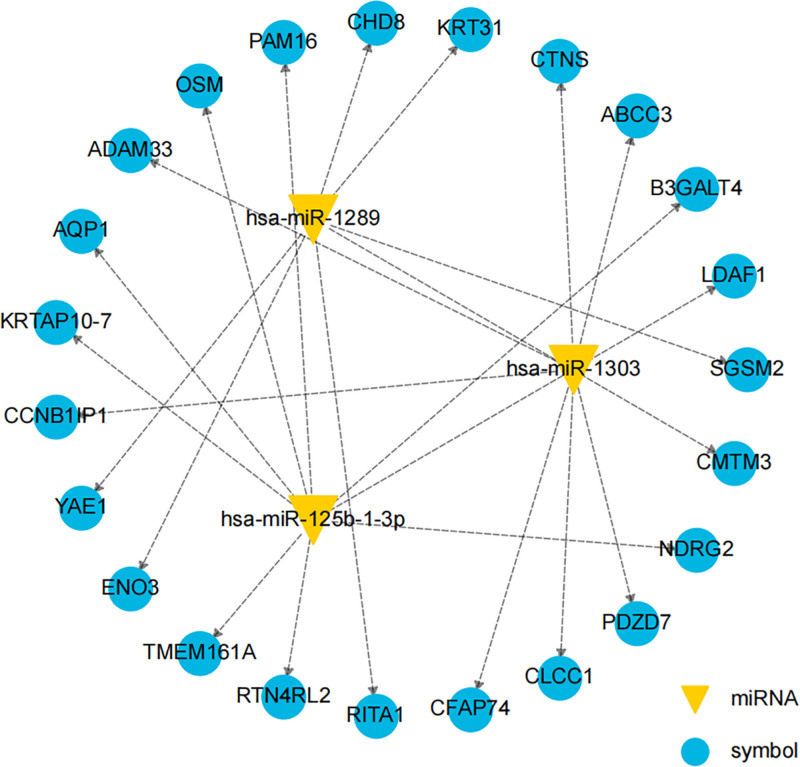
Target genes for hsa-miR-1303, hsa-miR-125b-1-3p, and hsa-miR-1289. The yellow triangles represent miRNAs, surrounded by blue circles represent target genes, and arrows represent miRNAs targeting these genes.

Target genes for differentially expressed hsa-miR-1303, hsa-miR-125b-1-3p, and hsa-miR-1289 were predicted using the Miranda and RNAhybrid algorithms, respectively. Their intersection was taken as the final result, duplicates were removed, and the top 23 genes with the highest interaction numbers were selected at a significance level of *P* < .05. We constructed an miRNA–target gene network centered on miRNA targets (Fig. 4).

### 3.5. Gene Ontology (GO) enrichment of differential genes

**Figure 5.**
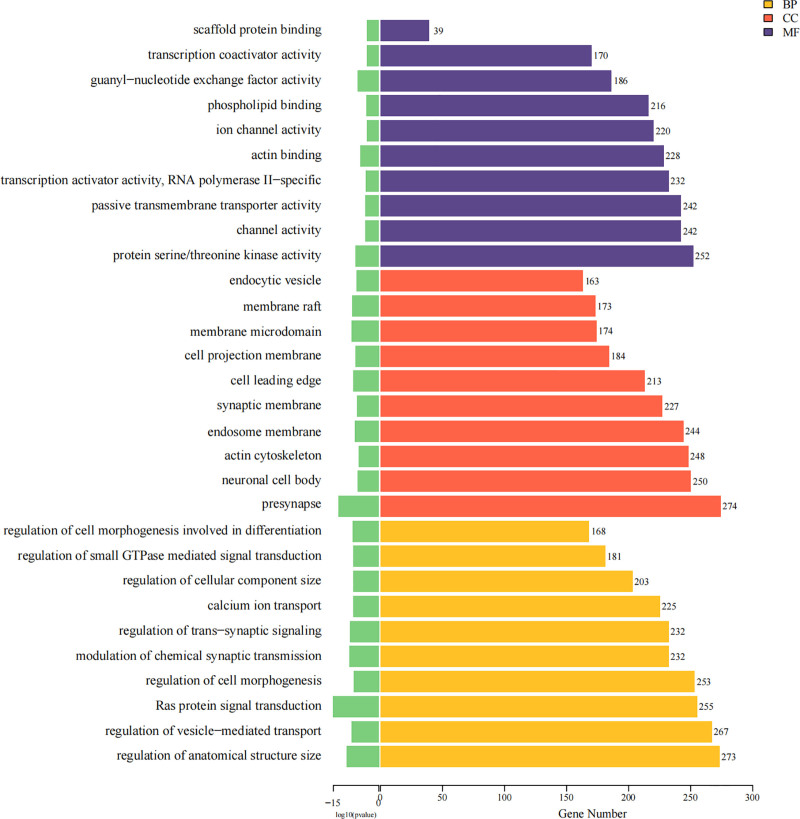
GO enrichment analysis of differential genes. Purplish denotes molecular function, red denotes cellular component, and yellow denotes biological process, respectively. Numbers represent the distribution of the enriched target genes. Green denotes *P*-value, and the longer the *P*-value lines in the graph, the smaller and more significant the *P*-values are.

All target genes of differentially expressed miRNAs marked up or down in the results of differential expression analysis were analyzed using the R package clusterProfiler for GO enrichment analysis. GO analysis yielded 6466 functional enrichment datasets, including 2289 clustered in BP, 2150 in cellular components (CC), and 2027 in molecular functions (MF). The 10 GO functions displaying the most significant enrichment (*P*-value level) were selected from the BP, CC, and MF categories. The MF category included scaffold protein binding, transcription coactivator activity, guanyl-nucleotide exchange factor activity, phospholipid binding, ion channel activity, actin binding, transcription activator activity (RNA polymerase II-specific), passive transmembrane transporter activity, channel activity, and protein serine/threonine kinase activity. The CC category includes endocytic vesicles, membrane rafts, membrane microdomains, cell projection membranes, cell-leading edges, synaptic membranes, endosome membranes, actin cytoskeleton, neuronal cell bodies, and presynapses. In the BP category, the regulation of cell morphogenesis involved differentiation, regulation of small GTPase-mediated signal transduction, regulation of CC size, calcium ion transport, regulation of trans-synaptic signaling, modulation of chemical synaptic transmission, regulation of cell morphogenesis, Ras protein signal transduction, regulation of vesicle-mediated transport, and regulation of anatomical structure size (Fig. 5).

### 3.6. KEEG pathway enrichment analysis

**Figure 6.**
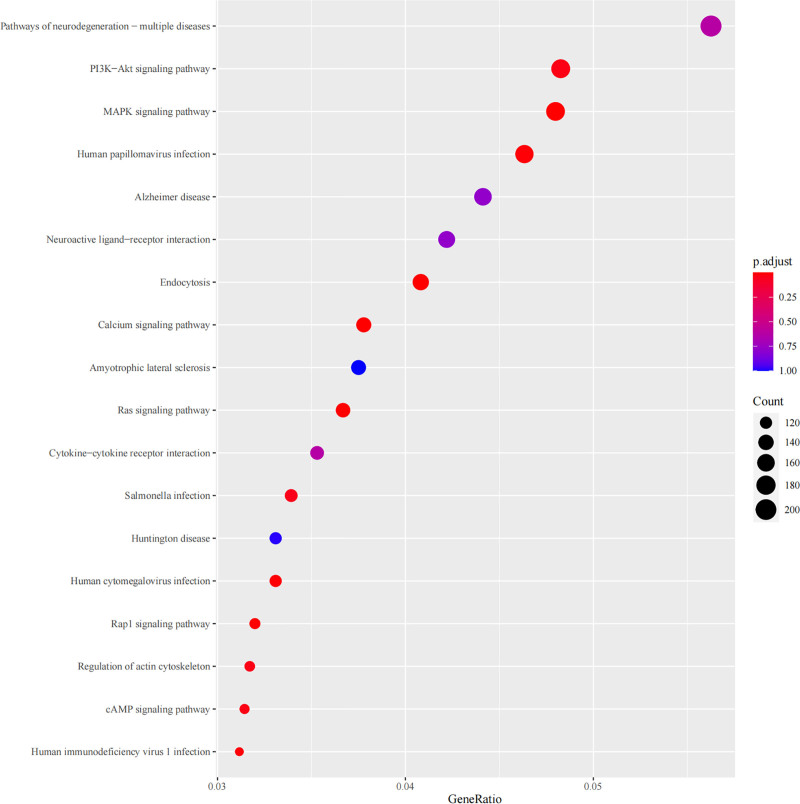
Results plot of KEGG enrichment analysis for differential genes. The horizontal axis is GeneRatio, representing the ratio of the number of miRNAs differentially expressed in the pathway entry to the total number of differentially expressed genes.

Similarly, Kyoto Encyclopedia of Genes and Genomes (KEGG) pathway enrichment analysis was performed on the differentially expressed genes using the R package cluster Profiler. The top 18 pathways with the largest number of KEGG entries were selected for analysis, and the related pathways were mainly concentrated in the PI3K-AKt, mitogen-activated protein kinase (MAPK), calcium, Ras, Rap 1, and cAMP signaling pathways (Fig. 6).

### 3.7. Prediction of the downstream target genes of hsa-miR-1303, hsa-miR-125b-1-3p, and hsa-miR-1289

**Figure 7.**
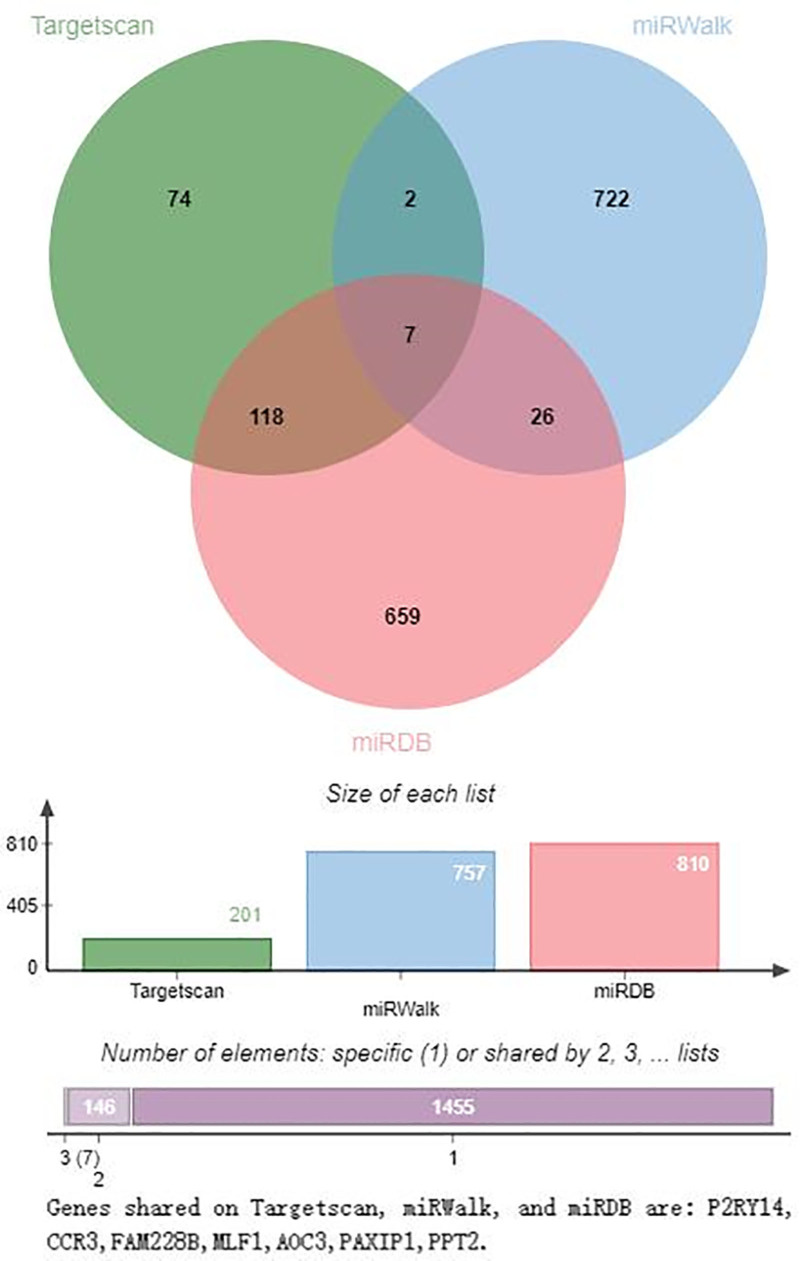
Venn diagram of the prediction of downstream target genes of hsa-miR-1303 on TargetScan, miRWalk, and miRDB. In this Venn diagram, the target genes of TargetScan, miRWalk, and miRDB are P2RY14, CCR3, FAM228B, MLF1, AOC3, PAXIP1, and PPT2. There are 7 in total.

**Figure 8.**
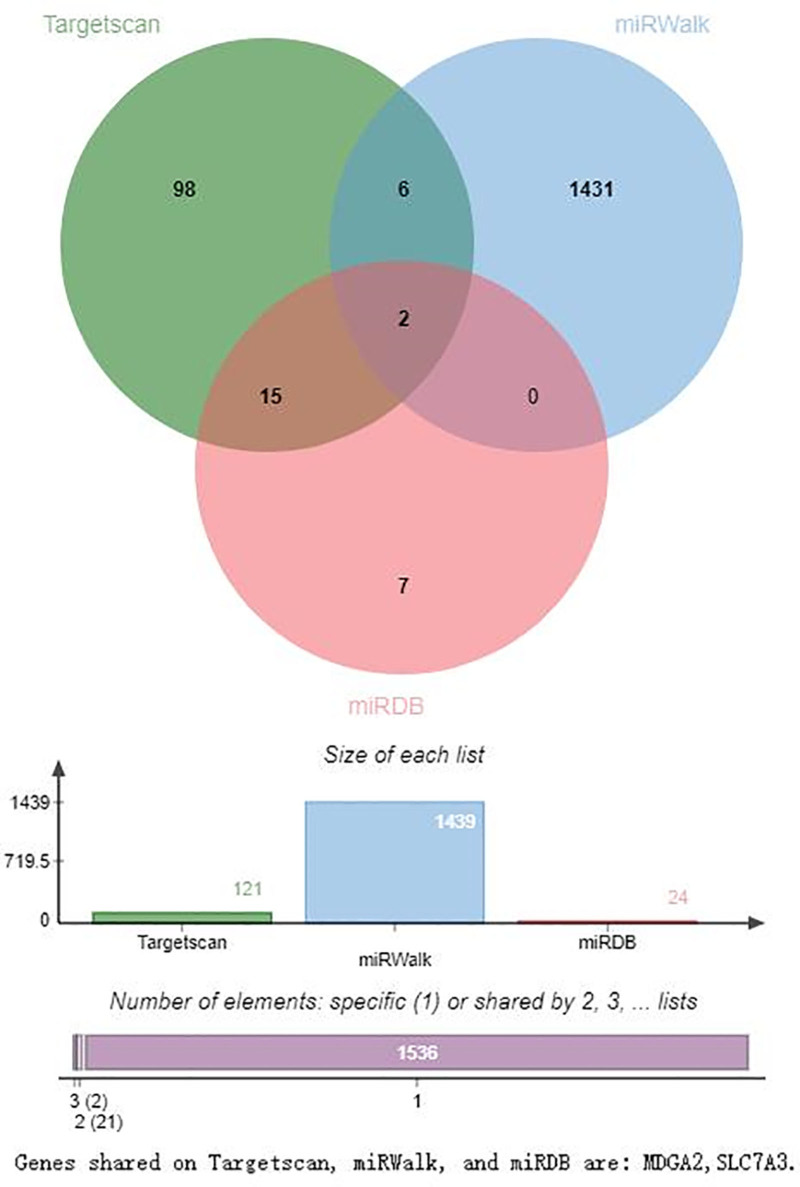
Prediction of the downstream target genes of hsa-miR-125b-1-3p on TargetScan, miRWalk, and miRDB. In this Venn diagram, only 2 genes, MDGA 2 and SLC7A3 are visible in the intersection of TargetScan, miRWalk, and miRDB.

**Figure 9.**
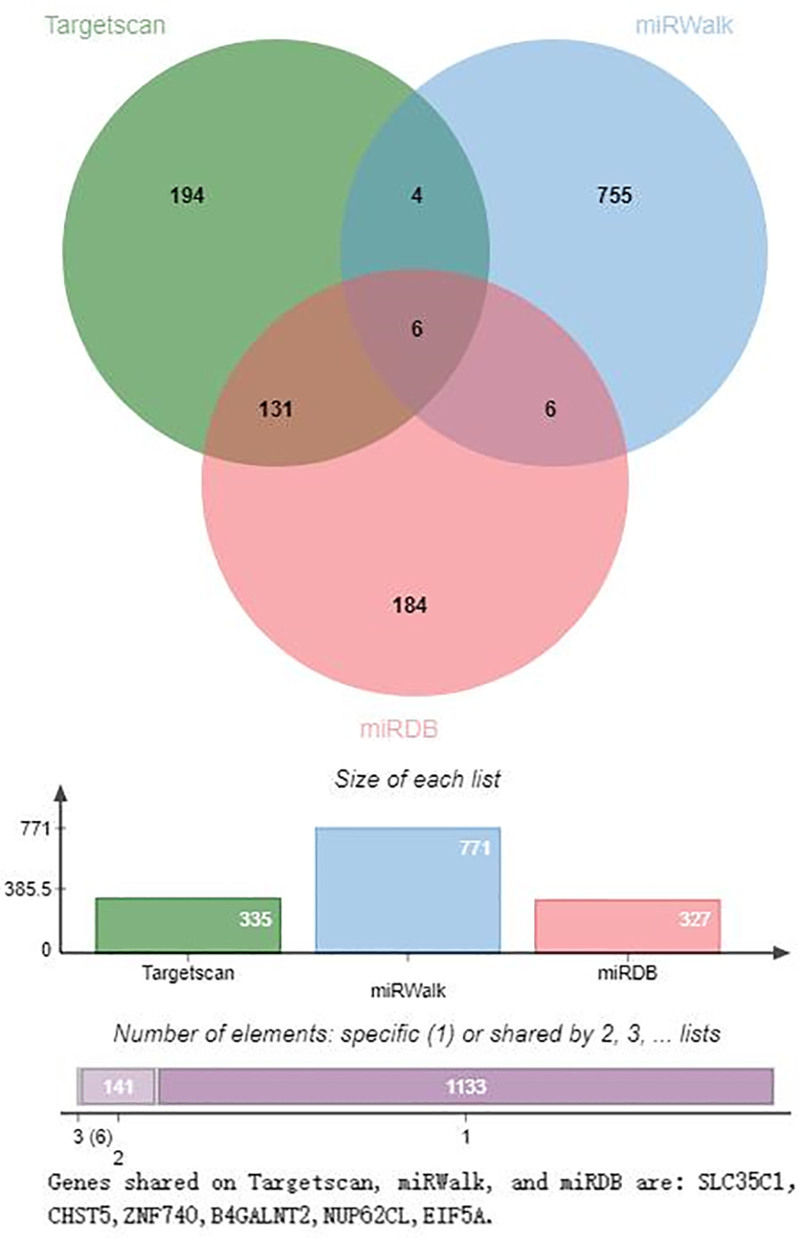
Venn diagram of the prediction of downstream target genes of hsa-miR-1289 on TargetScan, miRWalk, and miRDB. In this figure, it is seen that there are 6 genes: SLC35C1, CHST5, ZNF740, B4GALNT2, NUP62CL, and EIF5A in the intersection of TargetScan, miRWalk, and miRDB.

The downstream target genes of hsa-miR-1303, hsa-miR-125b-1-3p, and hsa-miR-1289 were predicted using TargetScan, miRWalk, and miRDB (Figs. 7–9). Figure 7 shows that the target genes of hsa-miR-1303 in TargetScan, miRWalk, and miRDB are P2RY14, CCR3, FAM228B, MLF1, AOC3, PAXIP1, and PPT2, respectively. There are 7 in all. As shown in Figure 8, only 2 genes, MDGA2 and SLC7A3, were visible at the intersection of TargetScan, miRWalk, and miRDB. As shown in Figure 9, 6 genes were identified at the intersection of TargetScan, miRWalk, and miRDB: SLC35C1, CHST5, ZNF740, B4GALNT2, NUP62CL, and EIF5A.

## 4. Discussion

The incidence of CIS is high in China. Middle-aged and elderly people are at high risk of CIS, and hypertension is the most important factor in its incidence. Long-term hypertension leads to hyaline changes in small blood vessels, lumen stenosis or occlusion, and subsequent ischemia and hypoxia in brain tissue, resulting in CIS.^[11,12]^ The incidence, case fatality rate, and disability rate of CIS are increasing annually.^[13]^ Currently, effective treatment in the acute phase of CIS is limited to cerebrovascular recanalization therapy, including intravenous thrombolysis and mechanical thrombectomy.^[14–16]^

Exosomes are cell-secreted lipid bilayer vesicles.^[3,4]^ In this study, the exosome diameter was observed to be approximately 50 to 150 nm by electron microscopy, all of which showed typical exosome biological properties. A comprehensive analysis of the expression profiles of miRNAs carried by exosomes from patients with CIS versus those from controls revealed significant differential expression. In the articles by Eyileten et al and Liang et al,^[17,18]^ it was noted that studies have shown several different miRNAs and their target genes are believed to be involved in the pathophysiology of IS. Some miRNAs can be used as diagnostic and prognostic biomarkers of CIS. The identification of 95 differentially expressed miRNAs (40 upregulated and 55 downregulated) revealed that hsa-miR-1303, hsa-miR-125b-1-3p, hsa-miR-100-5p, and hsa-miR-548ab were significantly upregulated, whereas hsa-miR-1289, hsa-miR-1185-1-3p, hsa-miR-10395-3p, hsa-miR-3173-3p, hsa-miR-654-5p, and hsa-miR-369-3p were significantly downregulated. These findings highlight the potential of these biomarkers and therapeutic targets.

Lewis et al suggested that miRNAs target many human genes, and more than one-third of human genes appear to be conserved miRNA targets.^[5]^ Functional clustering analysis revealed the biological role of these miRNAs. Hsa-miR-548ab was highly expressed in the CIS group, whereas hsa-miR-6873-3p and hsa-miR-605-3p exhibited varying levels of expression in the case and control groups. Similarly, unnamed miRNAs, such as chr9-56367, chrX-59355, and chr2-32448 were highly expressed in the case group, whereas chr12-13712 and chr16-20340 were more prominent in the control group. In this study, the GO analysis contained 6466 functionally enriched data points. Among these, the ten GO functions displaying the most significant enrichment were selected from the BP, CC, and MF categories. The top GO functions highlighted were essential roles in scaffold protein binding, transcription coactivator activity, ion channel activity, calcium ion transport, regulation of trans-synaptic signaling, modulation of chemical synaptic transmission, regulation of cell morphogenesis, and Ras protein signal transduction.

Target gene prediction using Miranda and RNAhybrid algorithms and the construction of an miRNA–target gene network enhances our understanding of regulatory networks involving these miRNAs. KEGG pathway enrichment analyses identified crucial BP and signaling pathways, such as the PI3K-Akt and MAPK pathways, which are known to play vital roles in cellular function and disease progression. This work indicates that the PI3K-Akt signaling pathway is not only a target of many cancers,^[19]^ but also participates in the improvement of cognitive function in CIS^[20]^ and the regulation of several other links of CIS. In addition, the MAPK signaling pathway is not only involved in the growth and differentiation of cancer cells^[21]^ and the development of the nervous system^[22]^ but may also participate in the regulation of multiple aspects of CIS.

Significantly differentially expressed hsa-miR-1303, hsa-miR-125b-1-3p, and hsa-miR-1289 have been investigated in various other diseases. For instance, the expression of hsa-miR-1303 is negatively correlated with the expression of GSK3β and SFRP1, which promote the proliferation of neuroblastoma by targeting GSK3β and SFRP1 in neuroblastoma tissues, and it may be a target for neuroblastoma therapy.^[23]^ The expression of miR-1303 is significantly upregulated in hepatocellular carcinoma, gastric cancer, and non-small cell lung cancer.^[24–26]^ hsa-miR-125b-1-3p is one of the miRNAs differentially expressed in the early stages of Alzheimer disease,^[27]^ and hsa-miR-1289 may be a tumor-suppressing miRNA in oral squamous cell carcinoma and non-small cell lung cancer.^[28,29]^ These findings indicated that these 3 miRNAs are involved in the occurrence and development of human tumors, CIS, and other diseases. The downstream target gene prediction results of hsa-miR-1303, hsa-miR-125b-1-3p, and hsa-miR-1289 in TargetScan, miRWalk, and miRDB were different from the target genes identified by the aforementioned researchers, suggesting that the genes and signaling pathways they may regulate differ across various diseases.

In this study, bioinformatic methods, such as functional clustering analysis and target gene prediction, were adopted to identify target genes, regulatory networks, and biomarkers related to CIS by the differential expression of miRNAs in the plasma of CIS patients, to provide new ideas for the diagnosis and treatment of CIS and provide a basis for further experimental verification of the interaction between miRNA and target genes.

The patients in this study were all from the Guangxi region of China, which indicates regional limitations. Further large-scale prospective cohort studies are required to determine whether these results are related to the local environment, dietary habits, or ethnic characteristics of the population. Additionally, the sample size of this study was relatively small, and the predicted differences in the expression and function of exosomal RNAs should be validated in larger clinical samples. Further cellular and animal studies are necessary to verify the expression of the downstream genes and their regulatory pathways.

## 5. Conclusion

The study comprehensively analyzed the microRNA expression profiles in CIS patients compared to controls, highlighting significant differential expression of microRNAs in plasma exosomes. These findings contribute to the growing body of knowledge on the role of exosomal microRNAs in CIS, offering potential biomarkers for diagnosis and targets for therapeutic intervention. Future studies should explore the functional implications of the identified miRNAs and their target genes in greater detail, paving the way for novel CIS management strategies.

## Acknowledgments

In addition to the support of these funds, we thank the Baise Key Laboratory for Research and Development on Clinical Molecular Diagnosis for High-Incidence Diseases for supporting this study.

## Author contributions

**Conceptualization:** Shengshan Yuan, Haifang Lan, Xuebin Li, Guijiang Wei.

**Data curation:** Shengshan Yuan, Haifang Lan, Yanlun Song, Gautam Agarwal, Xuebin Li, Guijiang Wei, Lina Liang.

**Formal analysis:** Xuebin Li, Guijiang Wei.

**Funding acquisition:** Xuebin Li, Guijiang Wei.

**Investigation:** Shengshan Yuan, Haifang Lan, Yanlun Song, Tingjun Liu, Gautam Agarwal, Xuebin Li, Guijiang Wei, Lina Liang.

**Methodology:** Shengshan Yuan, Haifang Lan, Yanlun Song, Tingjun Liu, Gautam Agarwal, Xuebin Li, Guijiang Wei, Lina Liang.

**Project administration:** Xuebin Li, Guijiang Wei.

**Resources:** Xuebin Li, Guijiang Wei.

**Supervision:** Xuebin Li, Guijiang Wei, Lina Liang.

**Software:** Haifang Lan.

**Validation:** Xuebin Li, Guijiang Wei.

**Writing – original draft:** Shengshan Yuan, Haifang Lan.

**Writing – review & editing:** Shengshan Yuan, Haifang Lan, Yanlun Song, Tingjun Liu, Gautam Agarwal, Xuebin Li, Guijiang Wei, Lina Liang.
